# Automatic Registration of TLS-TLS and TLS-MLS Point Clouds Using a Genetic Algorithm

**DOI:** 10.3390/s17091979

**Published:** 2017-08-29

**Authors:** Li Yan, Junxiang Tan, Hua Liu, Hong Xie, Changjun Chen

**Affiliations:** School of Geodesy and Geomatics, Wuhan University, Luoyu Road 129, Wuhan 430079, China; lyan@sgg.whu.edu.cn (L.Y.); liuhua@whu.edu.cn (H.L.); hxie@sgg.whu.edu.cn (H.X.)

**Keywords:** terrestrial LiDAR scanning, mobile LiDAR scanning, point cloud, registration, genetic algorithm

## Abstract

Registration of point clouds is a fundamental issue in Light Detection and Ranging (LiDAR) remote sensing because point clouds scanned from multiple scan stations or by different platforms need to be transformed to a uniform coordinate reference frame. This paper proposes an efficient registration method based on genetic algorithm (GA) for automatic alignment of two terrestrial LiDAR scanning (TLS) point clouds (TLS-TLS point clouds) and alignment between TLS and mobile LiDAR scanning (MLS) point clouds (TLS-MLS point clouds). The scanning station position acquired by the TLS built-in GPS and the quasi-horizontal orientation of the LiDAR sensor in data acquisition are used as constraints to narrow the search space in GA. A new fitness function to evaluate the solutions for GA, named as Normalized Sum of Matching Scores, is proposed for accurate registration. Our method is divided into five steps: selection of matching points, initialization of population, transformation of matching points, calculation of fitness values, and genetic operation. The method is verified using a TLS-TLS data set and a TLS-MLS data set. The experimental results indicate that the RMSE of registration of TLS-TLS point clouds is 3~5 mm, and that of TLS-MLS point clouds is 2~4 cm. The registration integrating the existing well-known ICP with GA is further proposed to accelerate the optimization and its optimizing time decreases by about 50%.

## 1. Introduction

Light detection and ranging (LiDAR) remote sensing technology has rapidly developed since it can collect 3D point clouds of object surfaces efficiently [1]. Terrestrial LiDAR scanning (TLS) with a LiDAR sensor mounted on a fixed platform [2] is widely used in various fields such as reverse engineering [3], cultural heritage documentation [4,5] and environmental monitoring [6,7,8]. Mobile LiDAR scanning (MLS) by integrating with several LiDAR sensors, a high-accuracy positioning and orientation system and a high-precision controlling system on a van or car provides a safer and more efficient way to capture large-scale geo-referenced point clouds [9,10]. It is being used at an increasing rate in the transportation industry [11,12], especially for road asset inventory [13,14] and in the production of high accuracy driving maps for intelligence driving [15].

The registration of point clouds is a fundamental issue in LiDAR remote sensing because point clouds are scanned from multiple scan stations or by different platforms, and they should be merged to obtain full coverage of a scene [16]. The aim of registration of different point clouds is to transform point clouds in different coordinate frames to a uniform coordinate reference frame. This paper deals with the registration problem of two TLS point clouds and the registration problem between TLS and MLS point clouds.

The traditional registration method is based on artificial markers that are placed in the scene during data acquisition. The positions of the markers must be manually extracted as tie points for registration. The marker-based registration is very reliable, but it requires very careful arrangements and is time-consuming [17]. Many automatic registration algorithms are proposed to improve the efficiency of the registration. The published methods can be classified into two categories: auxiliary data-based methods and 3D point-based methods [18]. Auxiliary data-based methods usually incorporate photographic images and point clouds for registration [19,20], or incorporate intensity images and point clouds for registration [21,22]. The image-assisted or intensity-assisted registration may bring up extra calibration of cameras and scanners, and the quality of the third-party data also has a direct effect on the registration process.

Many experts have directly used 3D point-based methods to solve the registration problem. The 3D point-based methods are usually divided into a coarse registration step and a fine registration step [23]. The feature-based algorithm is a common way to achieve coarse registration, which establishes correspondences between two point clouds using extracted features. The used features, such as geometric curvature, main frame and point signature, are invariant with rotation and translation [24,25]. Johnson and Hebert proposed a local shape descriptor called ‘spin image’ to match or recognize an object [26]. This histogram-feature registration is strongly affected by the given parameters—bin size, image width, and support angle. Rusu, et al. proposed a local non-linear optimizer called ‘sample consensus initial alignment’ (SAC-IA) to accomplish the registration, which employed their own fast point feature histogram (FPFH) as the feature descriptor [27]. Tombari, et al. and Hänsch, et al. reviewed the existing feature descriptors and compared their performance, and the results indicate that the specific method to extract features has to be carefully chosen [28,29]. It is hard to establish robust correspondences by the extracted features because of the uneven point density, clutters and outliers, repeated objects, sheer size, and partial overlap of the point clouds [17,30]. The optimization of feature-based registration also needs exhausted computation [31].

A well-known fine registration method is the iterative closest points (ICP) algorithm [32], where each point in a one point cloud is paired with the closest point in the other point cloud to form correspondences, and then a point-to-point error metric (the mean of the squared distances of the correspondences) is minimized. The process is iterated until the error becomes smaller than a threshold or the maximum iteration is achieved. Chen and Medioni proposed a point-to-plane error metric ICP [33], which is more accurate than the point-to-point ICP [34]. Hereafter, some modified ICP methods are proposed and they are distinct from four aspects [35]: (1) selection of candidate points; (2) search strategy of nearest points to establish correspondences; (3) weighting relationship of correspondences or rejection of invalid correspondences; and (4) error metric and optimization. Many ICP algorithms have been achieved in the Point Cloud Library (PCL) [36]. However, ICP is easy to fall into local optimum, which is inherently determined by the employed local optimizer [37]. Hence, their performance critically relies on the initialization quality (the quality of coarse registration), and only local optimality is guaranteed.

Unlike the above coarse-fine registration, registration based on genetic algorithm (GA) uses a global search strategy which automatically finds the optimal solution in the search space [38]. GA is a heuristic optimizer that simulates the evolution of nature—selection (survival of the fittest), crossover, and mutation. Jacq and Roux presented a framework of 3D medical point cloud registration based on GA [39]. Brunnstrom and Stoddart proposed a GA registration method for free-form surface matching for the first time, which achieved finding correspondences rather than searching optimal solutions in search space [40]. This method is not applicable when there are too many matching points. Yamany, et al. presented a GA registration method whose fitness function was to maximize the reciprocal of the sum of squared errors (SSE) between correspondences [41]. Silva, et al. gave more details of GA registration and proposed a better objective function to minimize the mean squared errors (MSE) between correspondences with outlier rejection [42,43]. Hereafter, the scholars also presented some different GA registration methods and their fitness functions were also based on the MSE [37,44,45]. MSE is originated from local optimization and it may not be globally optimal. The fitness values are often needed to scale into maximum values by a negative exponential function. In addition, GA registration is complicated and time-consuming because of the global search strategy of GA and the complexity of scanned point clouds.

This paper proposes an efficient GA registration method for registering two TLS point clouds or two point clouds where one is scanned by TLS and the other is scanned by MLS. In order to make the GA registration workable and improve the registration efficiency, the selection of matching points is first applied to eliminate the far, redundant and noisy points and to select partial points representing the main features before GA evolution. Besides, the scanning station position acquired by the TLS built-in GPS and the quasi-horizontal orientation of LiDAR sensor in data acquisition are used as constraints to narrow the search space in GA. Furthermore, the calculation of fitness values—the most time-consuming step of GA registration—is parallel-computed. Instead of the MSE-based fitness function, a new and more accurate fitness function, named ‘Normalized Sum of Matching Scores’ (NSMS), is proposed to evaluate the solutions.

The remainder of the paper is structured as follows. The GA is firstly introduced in Section 2. The proposed GA registration is described in Section 3. Then the experimental results and the discussions are described in Section 4, where the registration integrating ICP with GA is presented. The conclusions are described in the last section.

## 2. Genetic Algorithm

Genetic algorithm (GA) is a global and heuristic optimizer which simulates biological evolution. It maintains a population of candidate solutions and evolves by iteratively applying three genetic operators: selection (survival of the fittest), crossover, and mutation. In this section, the GA is briefly introduced. More details can be referred in [38,46,47].

In order to apply GA, the encoding mode should be first determined. Encoding is to present a solution in the search space as a chromosome that is composed of genes and can be computed by the genetic operation. The inverse mode is called decoding. The common numerical encoding methods are binary encoding and float encoding. The binary encoding is to convert a real to a binary and the corresponding decoding is to inversely transform a binary to a real. The float encoding directly uses a parameter as an encoding gene, which the genes do not need in order to be decoded. Float encoding is therefore more efficient in the optimization of multivariable functions and there is no conversion accuracy loss.

A standard GA is divided into three steps—initialization of population, calculation of fitness values, and genetic operation. A population is a set of chromosomes {***P***|***Ch***_1_, ***Ch***_2_, …}. In its initialization, a chromosome is often uniform-randomly generated in the search space. The pseudo code of initialization for float encoding is presented in Algorithm 1. The size of the population *M* is often empirically set to a few hundred. The search space is the solution domain of the optimization problem, which is defined between the negative and positive upper bound vector. Defining search space is a core issue in actual optimization.

**Algorithm 1.** The pseudo code of population initialization.1Input the upper bound vector of the solution 2domain *U* and population size *M*; 3For *I* = 1:*M*4 For *k* = 1:DIMENSION(*U*)5  Randomly generate *r* in [−*U_k_*, *U_k_*];6  *Ch_i_*_,*k*_ = *r*;7 End8End

The calculation of fitness values is based on a fitness function. The fitness function that is defined to evaluate the solutions is another core issue in actual optimization. It is scaled from the objective function of the optimization problem and provides the guidance for the GA selection operation. Therefore it directly affects the GA performance and should be carefully designed.

The genetic operation is a simulation of biological gene manipulation, including selection, crossover, and mutation. The selection operator is to select *M* chromosomes from the parent population for reproduction. The chance of selecting one chromosome as a father or mother should be proportional to the population size *M*. Remainder stochastic selection is a better method than the direct stochastic selection by fitness proportion. It can ensure the chromosomes with higher fitness proportions are chosen and have higher proportions in the selected population. Firstly, each chromosome in the population is copied several times for reproduction. The copied number of the *i*th chromosome is

(1) $$Num_{i}=\left\lfloor M\cdot F_{i}/\sum_{k}F_{k}\right\rfloor$$

where, *F_i_* is the fitness value of the *i*th chromosome. $\sum Num_{i}$ chromosomes are copied, and then the remained fitness value of the *i*th chromosome is

(2) $$F_{i}^{\prime }=F_{i}-\sum_{k}F_{k}\cdot Num_{i}/M$$

All remaining fitness values of the population are used to produce the other $M-\sum Num_{i}$ chromosomes by direct stochastic selection.

The crossover operator mates parents to produce two new offspring and the mutation operator alters one or more gene values in a chromosome from its initial state. To ensure that the genes of the optimal chromosome at each generation are not eliminated and damaged, the chromosome with the highest fitness is directly copied into the next generation. The crossover operator and mutation operator are dependent on the encoding. If the float encoding is applied, arithmetic crossover and non-uniform mutation are suitable for generating new chromosomes. Their pseudo codes are given in Algorithm 2. Here, two parameters, the crossover probability *P_c_* and mutation probability *P_m_*, should be set. Empirically, *P_c_* is 0.6~1 and *P_m_* is not more than 0.1.

**Algorithm 2.** The pseudo code of arithmetic crossover and non-uniform mutation.**arithmetic crossover****non-uniform mutation**1Input a mother chromosome *Ch*_1_ and1Input the upper bound vector of the solution2a father chromosome *Ch*_2_;2domain *U* and a chromosome *Ch*;3Randomly generate *p_c_* in [0, 1];3Randomly generate *p_m_* in [0, 1];4If *p_c_* < *P_c_*4If *p_m_* < *P_m_*5 For *k* = 1:DIMENSION(*Ch*_1_)5 *T* = (1 − *Current_g_*/*MAX_g_*)^2^;6  Randomly generate *r* in [0, 1];6 For *k* = 1:DIMENSION(*Ch*)7  *T_k_* = r*(*Ch*_2,*k*_−*Ch*_1,*k*_);7  Randomly generate *r* in [0, 1];8  *Ch*_1,_*_k_* = *Ch*_1,*k*_ + *T_k_*;8  If *r* > 0.59  *Ch*_2,*k*_ = *Ch*_1,*k*_ − *T_k_*;9   *Ch_k_* = *Ch_k_*+(*U_k_* − *Ch_k_*)*r*T;10 End10  Else11End11   *Ch_k_* = *Ch_k_* − (*U_k_* + *Ch_k_*)*r*T;

12  End

13 End

14End

The calculation of fitness values and genetic operation are iteratively applied, which constitutes the evolution of GA. The GA evolution is terminated when either of the following two criteria is satisfied:

- Maximum number of generations: exceeding the maximum generation *MAX_g_* makes the evolution stopped.
- Maximum number of generations that the best fitness keeps stable: if the number of generations, which the current best fitness value is unchanged, is equal to a given value *MAX_b_*, the evolution is also stopped. To find out the globally optimal solution, *MAX_b_* should not be too small.

## 3. Proposed GA Registration

### 3.1. Framework

Given the source point cloud ***S*** with points ***S****_i_* ∈ ***S*** and the target point cloud ***T*** with points ***T****_j_* ∈ ***T***, the registration problem is to find correspondences (***S****_i_*, ***T****_j_*) between ***S*** and ***T***, and to estimate the rigid transformation [36]

(3) $$T_{j}=t+RS_{i}$$

where, ***t*** = [*t_x_*, *t_y_*, *t_z_*]*^T^* is the unknown translation vector; ***R*** is the unknown rotation matrix that is expressed by a function of three Euler rotating angles *α*, *β*, *γ* around *x*, *y*, *z*-axes.

**Figure 1:**
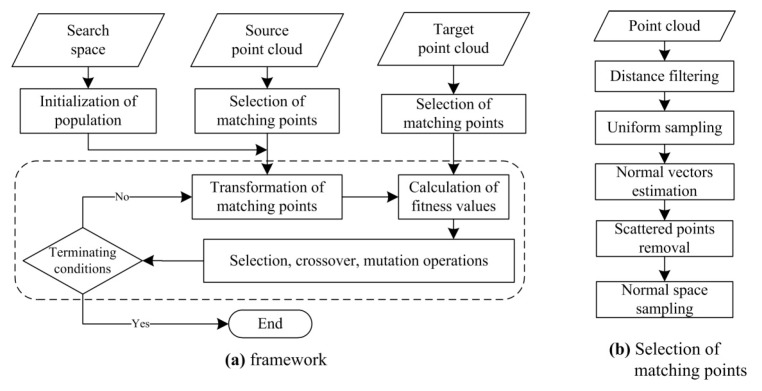
The framework and selection of matching points of the proposed GA registration. The GA evolution is showed in the dashed box. (**a**) The flow chart of GA registration; (**b**) The process of selection of mathing points.

The pipeline of the proposed GA registration to estimate optimal transformation parameters is divided into five steps—selection of matching points, initialization of population, transformation of matching points, calculation of fitness values, and genetic operation. This framework is illustrated in Figure 1a. As illustrated in Figure 1b, the selection of matching points includes five sub-steps—distance filtering, uniform sampling, normal vectors estimation, scattered points removal, and normal space sampling. Its purpose is to make the GA registration efficient by eliminating the far, redundant, noisy points and by selecting some percentage of points that can express the main features. Its details are presented in Section 3.2.

To further understand the proposed method, the pseudo code of the GA registration is given in Algorithm 3. The GA evolution consists of the steps from line 8 to line 27 in Algorithm 3 (the steps from 2 to 5 in the pipeline). Because the optimization contains multiple unknown parameters, the float encoding for GA is better compared with binary encoding. Each chromosome in the population is a six-dimensional vector ***Ch*** = [*α*, *β*, *γ*, *x*, *y*, *z*]. Before GA evolution, the population must be initialized. The pseudo code of “Initialization of Population” is shown in Algorithm 1.

The “Transformation” using Equation Equation (3) is conducted for each chromosome, and then the closest points of the transformed ***S*** in ***T*** are searched and the chromosomes are evaluated by a fitness function in “Calculation of Fitness”. According to the computed fitness values, the GA operations can be implemented to produce a new population. The GA evolution is stopped until the termination conditions are satisfied. The “Selection Operation” using the remainder of the stochastic selection has been described in Section 2. The float encoding is applied, so the arithmetic crossover and non-uniform mutation operation (Algorithm 2) are suitable for generating new chromosomes.

The “Calculation of Fitness” is the most time-consuming step of the GA registration because of the nearest neighbor searching between ***S*** and ***T***. The “Transformation” and “Calculation of Fitness” are independently conducted for each chromosome in the population. Therefore, these two steps can be accelerated by multi-thread parallel computing technology. In our program, the Open Multi-Processing (OpenMP) [48] is utilized to accelerate the computation.

**Algorithm 3.** The pseudo code of the Genetic Algorithm (GA) registration.1Input *S* and *T*; Input the upper bound15 If cur_best = last_best2vector *U* ;Input the given parameters;16  Iter_best = Iter_best + 1;3Selection Of Matching Points (*S*);17 Else4Selection Of Matching Points (*T*);18  Iter_best = 0;5Initialization of Population (*P*);19  last_best = cur_best;6Iter = 0; Iter_best = 0; cur_best = 0.0;20 End7last_best = 0.0; //_best means best fitness21 Foreach *Ch_i_* and *Ch_i+_*_1_ in *P*8While (Iter < *Max_g_* and Iter_best < *Max_b_*)22  Crossover Operation (*Ch_i_, Ch_i+_*_1_);9  Foreach *Ch_i_* in *P*23 End10   Transformation (*S*);24 Foreach *Ch_i_* in *P*11   *F_i_* = Calculation Of Fitness (*S*,*T*);25  Mutation Operation (*Ch_i_*);12  End26 End13  cur_best = Selection Operation (*P*);27End //While14  Iter = Iter + 1;



To solve the GA registration, two core issues should be defined—(1) search space (solution domain); and (2) a fitness function to evaluate the solutions. The search space is expressed in Section 3.3, where the auxiliary constraints of the scanning station are implemented to narrow the search space. The proposed NSMS fitness function is presented in Section 3.4.

### 3.2. Selection of Matching Points

A large volume of noisy and unevenly distributed point clouds make the registration computation expensive. This section introduces some methods for selecting the candidate points for matching. The purpose is to make the registration efficient by eliminating the far, redundant, noisy points and then selecting some percentage of points that can still express the main features.

The points far away from the scanners are sparse and have significant noise. Hence, they are first eliminated by distance filtering. The max distance threshold *D_max_* for distance filtering is set according to the specifications of the scanning system. The *D_max_* should not be larger than the max range and can be often set to near the effective range of the scanner. e.g., under Riegl test conditions, the Riegl VZ400’s max range with target reflectivity 10% is 120 m and its accuracy is 5 mm when the range is 100 m [49], so the *D_max_* can be set to 100 m.

After distance filtering, uniform sampling is applied to avoid the near-field bias inherent in regular angular sampling and to achieve a reduction of the point count by voxel grid filtering [31]. The voxel grid filtering is that the points in a grid are replaced with the nearest point of the grid center. Then the normal vectors and curvatures are estimated by a local covariance matrix algorithm [50]. The voxel size *V_g_* for uniform sampling can be set several times that of the point resolution. The vertical resolution can be computed by the vertical angular step width of the scanner, and the horizontal resolution can be calculated by the horizontal angular step width in TLS or the scan speed and the driving speed in MLS. e.g., if the angular step is *θ* rad, the resolution at scanning distance *D* is *θ* × *D*; if the scan speed is *L* lines/sec and the driving speed is *V*, the horizontal resolution (scan line spacing) of MLS point cloud is *V*/*L*. Obviously, it is difficult to assign the *V_g_* to a certain value because the resolution is changed with the scanning distance. In order not to affect the registration accuracy as much as possible, the *V_g_* should be set as small as possible.

**Figure 2:**
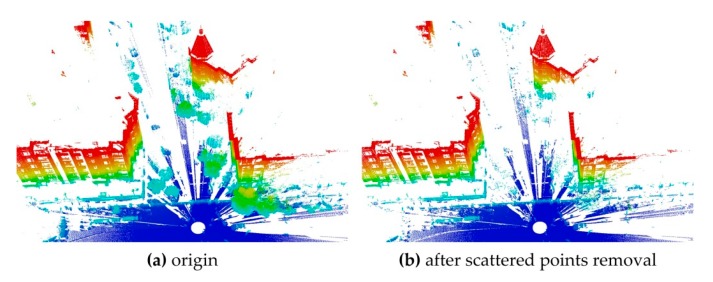
Scattered points removal. The point clouds are rendered by elevation. (**a**) The point cloud before scattered points removal; (**b**) the point cloud after scattered points removal.

The point clouds contain many outliers and tree leave points. The tree leaves are easy to shake with the wind. The outliers and tree leave points will affect the registration. They should be removed and the point count can be further reduced. These points are located in irregular shapes and are scattered (i.e., scattered points), so their curvatures are often larger than those of most other man-made objects (i.e., smooth points). It has been found experimentally that most of the scattered points can be removed by curvature filtering. The points whose curvatures are larger than a threshold *C* are regarded as scattered points. When *C* is set to 0.05, most scattered points can be removed (Figure 2).

**Figure 3:**
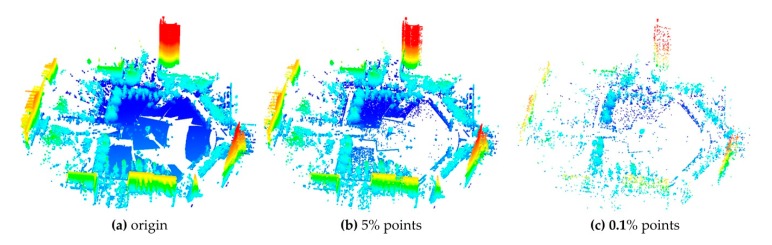
Normal space sampling. The point clouds are rendered by elevation. (**a**) The point cloud before normal space sampling; (**b**) the point cloud after 5% points were selected; (**c**) the point cloud after 0.1% points were selected.

After the above steps, there may be still plenty of points. A normal space sampling strategy [35] is employed to select *p_S_* points from a source point cloud and *p_T_* points from a target point cloud as candidate matching points for registration. In tests of the well-known ICP registration [35], the normal space sampling is proved to be superior compared with random sampling. Figure 3 gives an example of the normal space sampling. It can be seen that this algorithm is able to maintain the main features when the sampling ratio is small. The sampling ratio is crucial to improvement of registration efficiency but it would affect the registration accuracy if the ratio is too small. It can be tested in actual registration.

### 3.3. Search Space

The GA registration is a process of evolution which finds the optimal solution in the search space. The search space of point cloud registration is defined between the negative and positive six-dimensional upper bound vector of the six transformation parameters. The upper bounds of *α*, *β*, *γ* are all 180° and the upper bounds of *t_x_*, *t_y_*, *t_z_* are unlimited when they are not constrained. It is a very large search space. If the search space is too large, the registration may become slow or may be easily premature, or even degenerate into a random search process. Hence, getting a limited search space is important.

In the TLS-TLS and TLS-MLS registration, the prior information of the point clouds is used to limit the search space. The MLS point clouds are directly geo-referenced through the high-accuracy positioning and orientation system (e.g., a GPS/INS system) [10], so they are located in the uniform geodetic coordinate reference frame. However, the TLS point clouds are located in local scanner coordinate systems. To narrow the search space, the position of the scan station acquired by TLS built-in GPS and the quasi-horizontal setup of TLS are used as constraints in our method.

If a built-in GPS is installed in the TLS laser scanner, the position of the scan station can be acquired by the built-in GPS in the field work. There is not much information about the specifications of the built-in GPS. What can be known is that the positioning mode of the built-in GPS is point positioning, whose accuracy is meter-level or even decimetre-level [51], so the upper bounds of *t_x_*, *t_y_*, *t_z_* can be set to 10 m. During scanning, the scanners are usually placed near horizontally, so *α*, *β* are close to 0°. Taking into account the actual errors, their upper bounds are set to 5°. Therefore, the search space is set to (*α*, *β*, *γ*, *t_x_*, *t_y_*, *t_z_* |*α*, *β* ∈ [−5°, 5°], *γ* ∈ [−180°, 180°], *t_x_*, *t_y_*, *t_z_*∈ [−10 m, 10 m]), i.e., the upper bound vector *U* is (5,5,180,10,10,10).

The used TLS laser scanner may not provide a built-in GPS. Under this circumstance, the other auxiliary measurements of the scan station are indispensable—e.g., measuring by an external GPS RTK is a good choice. The GPS RTK—the accuracy of which is centimetre-level—is more accurate than the point positioning mode [51], and then the upper bounds of *t_x_*, *t_y_*, *t_z_* can be set to 0.1 m. The search space for GPS RTK is 1 million times less than that for built-in GPS. Other measuring methods—for example by a Total Station with millimeter-level accuracy—can also be implemented in the field work if necessary. Either way, the upper bounds of *t_x_*, *t_y_*, *t_z_* can be set on the basis of the accuracy level of the auxiliary measurements.

### 3.4. Proposed NSMS Fitness Function

A fitness function defined to evaluate the solution domain provides the guidance for the GA selection operation. The proposed NSMS fitness function is presented here.

Given correspondences between *S* and *T*, a common way to estimate the transformation parameters is to minimize the error metric *E* between all selected correspondences.

(4) $$E=\frac{1}{N}\sum_{i=1}^{N}d_{i},\;d_{i}=\left\|T_{j}-\left(t+RS_{i}\right)\right\|$$

where, *N* is the number of correspondences.

In the traditional GA registration method, the error metric is required to convert into a maximum form to define the fitness function. The negative exponential function is a common way to convert the objective function to fitness function

(5) $$F=e^{-E}$$

where, ***F*** is the fitness value.

Differently, the NSMS fitness function is proposed here to avoid the conversion from *E* to *F* and to evaluate the solutions accurately. It is expressed as

(6) $$F=\frac{1}{N}\sum_{i=1}^{N}Sc\left(d_{i}\right)$$

where, *Sc* is a score function of *d_i_*, which must be satisfied with two criteria: (1) 0 < *Sc* ≤ 1; (2) *Sc* is monotonically decreasing. The first criteria is to make the fitness function is optimal even if *d_i_* is larger than 1; the second criteria is utilized to directly access to the maximum *F*.

For partially overlapped point cloud registration, a continuous score function satisfied with the above criteria is designed

(7) $$Sc\left(d_{i}\right)=\{\begin{array}{cc} \exp\left(\log\left(Sc_{ideal}\right)\frac{d_{i}}{d_{ideal}}\right) & 0\leq d_{i}\leq d_{ideal}\\ Sc\cdot \exp\left(\left(\log\frac{Sc}{Sc_{ideal}}\right)\frac{d_{i}-d}{d-d_{i}}\right) & d_{ideal}<d_{i}\leq d\\ Sc & d_{i}>d \end{array}$$

where:

*d* is the distance threshold that is applied to separate the correspondences into two parts: Inliers in the overlap area and outliers in the non-overlap area;
*d_ideal_* is the ideal distance that is applied to further separate the correspondences in the overlap area into two parts: correspondences in the ideal area and correspondences in the buffer area;
*Sc* is the score of *d*; and *Sc_ideal_* is the score of *d_ideal_*.

**Figure 4:**
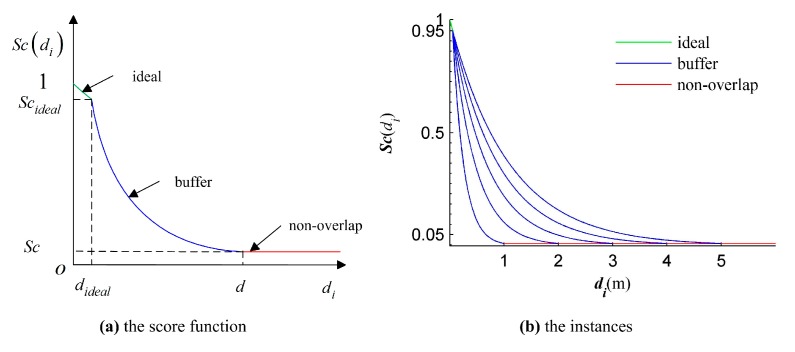
The score function and its instances. (**a**) The diagram of score function; (**b**) the instances of score function under different distance thresholds.

The *NSMS* fitness function means that the optimization problem is to minimize the sum of the distances between correspondences and to maximize the number of inliers. Just as the negative exponential function is used to convert the objective function in the Equation (5), it is also used to define the score function. The score function and its applied instances are illustrated in Figure 4.

Four parameters should be given for the score function. The *Sc* is set to a low confidence level value 0.05 and the *Sc_ideal_* is set to a high confidence level value 0.95. That is to say, the higher the score, the greater the probability that the correspondence belongs to the inliers. The *d_ideal_* need to be set to a small value to ensure that the correspondences with small distances are given high scores. In the registration, the *d_ideal_* is set to 5 cm. The *d* is determined by the attributes of score function. It is shown in Figure 4b that the smaller the *d*, the steeper the curve; the bigger the *d*, the flatter the curve. Hence, the smaller the *d*, the fewer the solutions with high fitness values, and then the more difficult it is to find out the globally optimal solution; the bigger the *d*, the closer the scores of inliers and outliers, and the more inaccurate the final solution. In the proposed GA registration, the search space is limited by auxiliary constraints of the scanner station (described in Section 3.3), and so the *d* can be intermediately set to 2 m.

## 4. Results and Discussions

### 4.1. Test Datasets

**Figure 5:**
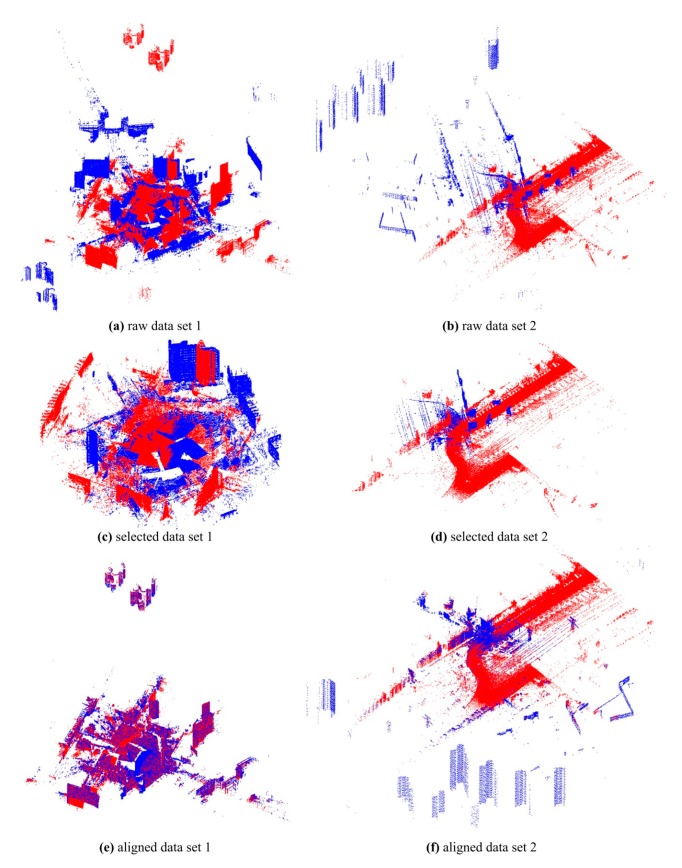
The test datasets. The red data are target point clouds and the blue data are source point clouds. (**a**) The raw point clouds of data set 1; (**b**) the raw point clouds of data set 2; (**c**) the point clouds of data set 1 after selection of matching points; (**d**) the point clouds of data set 2 after selection of matching points; (**e**) the registered point clouds of data set 1; (**f**) the registered point clouds of data set 2.

Two datasets were scanned to validate the effectiveness of the proposed GA registration. Each dataset contains two point clouds: a source point cloud and a target point cloud. The dataset 1 as shown in Figure 5a was scanned by a TLS 3D Riegl VZ400 scanner. The number of points in each point cloud is about one hundred million. The station positions providing constraints of the search space were measured by the built-in GPS of the scanner. The important specifications of the scanner from Riegl’s official website [49] are displayed in Table 1. The horizontal and vertical scan angular step widths are selectable. Towards the dataset 1, both were set to 0.015° before scanning. The resolution of the point clouds at distance *D* is about 0.015 × π/180 × *D* (2.5 cm@100 m).

The dataset 2 as shown in Figure 5b includes a source point cloud scanned by the Riegl VZ400 scanner and a target point cloud scanned by MLS. The number of points in each point cloud is about ten million. The positioning vector of the source point cloud for GA registration was also measured by the built-in GPS of the Riegl VZ400. The horizontal and vertical scan angular step widths of the Riegl VZ400 were set to 0.15° before scanning. The resolution of the source point clouds at distance *D* was about 0.15 × π/180 × *D* (5.2 cm@20 m). The MLS system contains a 2D Riegl VUX scanner. Its important specifications from Riegl’s official website are displayed in Table 1. The scan angular step width and the scan speed are selectable. Towards the MLS system, they were set to 0.5° and 200 scan/s respectively. The vertical resolution of the target point cloud at distance *D* was about 0.5 × π/180 × *D* (5.2 cm@10 m). During scanning, the driving speed was approximately 40–45 km/h, and then the horizontal resolution (scan line space) of the target point cloud was about 5–6 cm. The comparison of the two datasets is displayed in Table 2.

**Figure 6:**
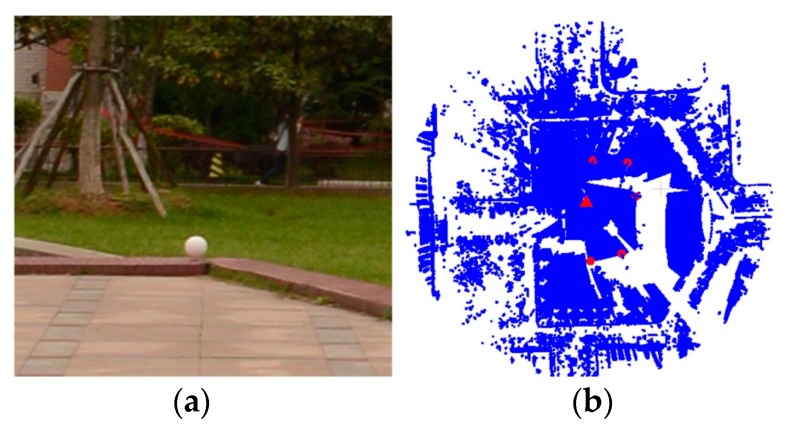
The spherical targets of data set 1. (**a**) the used target (white sphere). (**b**) the distribution of the targets (red circle points). The red triangle denotes the scan station.

**Figure 7:**
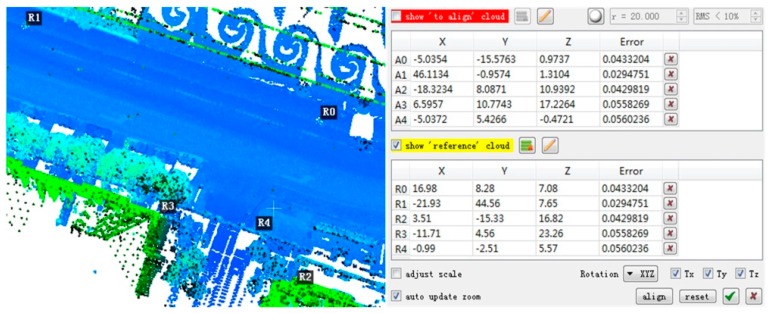
The rough matching for dataset 2 by software Cloud Compare.

To measure the registration accuracy quantitatively, the root mean square error (RMSE) between the ***S*** and its reference was calculated. The reference of the dataset 1 was computed by 5 spherical targets (Figure 6) in the scene. The distances from the targets to the scan station are between 20 m and 40 m. The center for each target was extracted. The center point pairs of the source and target point clouds were registered and then the source point cloud was transformed to form the reference. The extraction and transformation were operated on the Riegl’s software RiSCAN PRO [49]. The reference of the dataset 2 was estimated by artificial rough matching (Figure 7) and then the fine ICP registration. 5 point pairs were manually selected for rough matching. The rough matching was operated on the open source software CloudCompare [52] and then the ICP registration was computed by the open source Point Cloud Library (PCL) [36].

### 4.2. Evaluation of the Proposed GA Registration

The algorithms were programmed using the C++ language and the verification was operated on a computer with Intel Core i7-4790 CPU @ 3.60 GHz, 8 G memory and quad-core processors. Since GA is a stochastic optimizer and the matching points are randomly selected, 50 experiments were carried out for each test. The test was considered a failure if the RMSE was larger than 10 cm. The failure rate was computed to evaluate whether the registration is robust.

The running time of the GA registration mainly consists of two parts—the running time of selection of matching points and the optimizing time of GA evolution. The selection of matching points is a pre-processing step. Without the normal space sampling in the preprocessing, it is the same for all tests of a data set. Hence, the optimizing time of the GA evolution indicates the efficiency of the GA registration. The number of generations (iterations) of the GA evolution was also used to evaluate the efficiency.

The specified algorithm parameters of the evaluation are given in Table 3, in which the first row is the parameters of the selection of matching points, and the second row is the parameters of the GA evolution. As described in Section 3.2, the *D_max_* was set to near the effective range of the scanner (Table 1) and the *C* was set by experiments. According to Table 2, the *V_g_* was set near to the point resolution of dataset 1 with the range at *D_max_*. The *p_T_* was set to 5% which could ensure enough object features for registration (Figure 3b), and the *p_S_* effect on the efficiency was tested. The parameters of the GA evolution were set empirically as described in Section 2. The parameters of the fitness function not included in Table 3 were *d* = 2 m, *d_ideal_* = 0.05 m, *Sc* = 0.05, and *Sc_ideal_* = 0.95 (Section 3.4).

**Figure 8:**
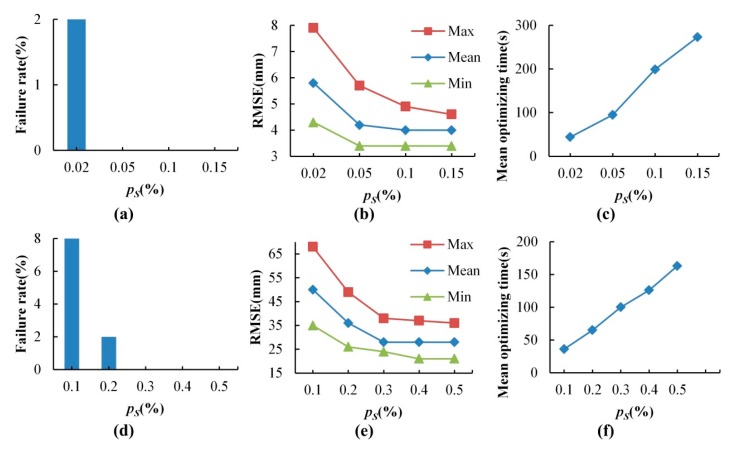
The failure rates, RMSEs and mean optimizing time of different sampling ratios. (**a**–**c**) are the results of data set 1. (**d**–**f**) are the results of data set 2.

**Figure 9:**
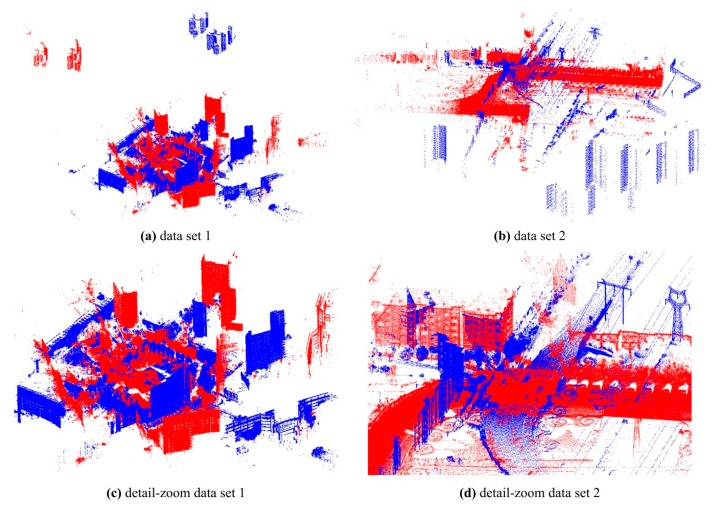
The examples of the situations where the registration goes wrong. The red data are target point clouds and the blue data are source point clouds. (**a**) The mismathed point clouds of data set 1; (**b**) the mismathed point clouds of data set 2; (**c**) a detail-zoom of (**a**); (**d**) a detail-zoom of (**b**).

The results of selection of matching points without normal space sampling are illustrated in Table 4 and Figure 5c,d. It took about 255 s for dataset 1 and 51 s for dataset 2. It can be seen that many noisy, redundant and scattered points were removed and the point count was greatly reduced while the main parts of the point clouds were retained. The failure rates, RMSEs and mean optimizing times in different sampling ratio cases are shown in Figure 8. The results showed in Figure 8 indicate that the increase of *p_S_* causes the decrease of failure rate and the RMSE. Through manual inspection, the failures were really the situations where the registration goes wrong as shown in Figure 9. The optimizing time is linear complexity with the sampling ratio. For dataset 1, when *p_S_* was more than 0.1%, the failure rate was 0 and the RMSE became unchanged, so *p_S_* was set to 0.1% in the subsequent experiments and results. Similarly, the *p_S_* of dataset 2 was set to 0.5% in the subsequent experiments and results.

**Figure 10:**
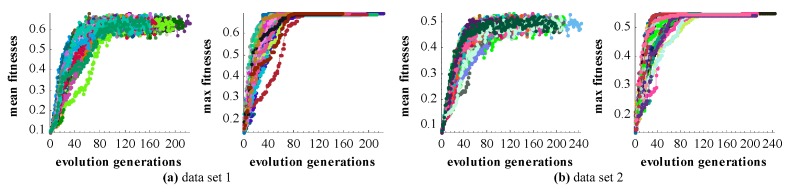
The mean and maximum fitness values of GA. A polyline with random color represents the change of the fitness value of one experiment. (**a**) The change of the fitness values of data set 1; (**b**) the change of the fitness values of data set 2.

The proposed GA registration can accurately align the laser scanning point clouds, where the registration accuracy of dataset 1 is 3~5 mm and that of data set 2 is 2~4 cm. The accuracy of dataset 2 is lower one order of magnitude than that of the data set 1. The main reason is that the target point cloud of data set 2 is scanned by MLS and it is much noisier than the TLS point clouds. The aligned datasets are qualitatively shown in Figure 5e,f. Additionally, the mean and maximum fitnesses of GA evolution are shown in Figure 10. It can be seen that the number of evolution generations for the flat parts of the fitness curves is more than half of the total number of evolution generations. It means that the GA’s local convergence rate is slow.

### 4.3. Comparative Study of the Fitness Functions

A study was conducted to compare our proposed NSMS fitness function with the published Silva fitness function [43]. The Silva fitness function is a typical MSE-based fitness function as given in Equation (5), which is converted from the objective function as given in Equation (4). Differently, the NSMS fitness function is directly mapped from the distances of the correspondences. In Silva fitness function, the distance threshold *d* like the algorithm parameter of the score function in the Equation Equation (7) is also applied to separate the correspondences into two parts:

(8) $$d_{i}=\{\begin{array}{cc} d_{i} & d_{i}\leq d\\ d & d_{i}>d \end{array}$$

In the comparative test, the sampling ratio *p_S_* was set to 0.1%, 0.5% for the dataset 1 and dataset 2 respectively according to the experimental results displayed in Figure 8, and the other algorithm parameters were the same as the parameters used in Section 4.2. 50 experiments were also carried out. The accuracy and efficiency of the Silva and NSMS fitness function for proposed GA registration are listed in Table 5. The results show that the NSMS fitness function was more accurate and efficient than the Silva fitness function. The optimizing time of the NSMS fitness function was about 20% less than that of the Silva fitness function.

### 4.4. Registration Integrating ICP with GA

To accelerate the convergence of optimization, the ICP was integrated with GA for registration. The combined method is called GA + ICP here. The integrating strategy is that ICP is executed after GA evolves some generations and most of the individuals in the population are located in a narrowed search space. Because the fitness values in the narrowed search space were close, to control the evolution generations of GA, the second terminating condition of GA (Section 2) could be modified to “if the number of generations, which the difference between the best fitness of the current generation and that of the previous generation is less than a given minor number *e*, is equal to a given value *MAX_b_*, the evolution is also stopped”. *e* is the searching control parameter. This is the only changed content of GA.

**Figure 11:**
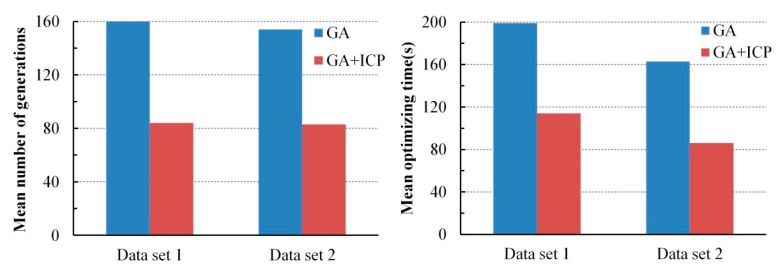
The mean number of generations and optimizing time of GA registration and GA + ICP.

The GA + ICP was tested, where *e* was set to 0.001 and 50 experiments were also carried out. The point-to-plane ICP [33] was applied. To reject invalid correspondences in ICP, the correspondence distance threshold was set to 0.2 m and the constraint normal angle is set to 10°. The results illustrated in Figure 11 indicate that the optimizing efficiency of GA + ICP increased by about 50% compared with the GA registration alone.

## 5. Conclusions

This paper proposes an accurate and efficient GA registration method for automatic alignment of two TLS point clouds or two point clouds scanned by TLS and MLS respectively. It is divided into five main steps: selection of matching points, initialization of the population, transformation of matching points, calculation of fitness values, and genetic operation. In order to make the GA registration workable, the aided localization and priori quasi-horizontal orientation of the scan station were used as constraints to narrow the search space. To get accurate results, a new fitness function named ‘normalized sum of matching scores’ (NSMS) is proposed to evaluate the solutions instead of the MSE-based fitness function. To improve the registration efficiency, the selection of matching points was first applied to eliminate the far, redundant and noisy points and to select partial points representing the main features before GA evolution. Besides, the calculation of fitness values, the most time-consuming step of GA evolution, was parallel-computed.

Two test datasets including a TLS-TLS data set and a TLS-MLS data set were scanned to validate the effectiveness of the proposed GA registration. The experimental results indicate that the RMSE of TLS point clouds registration is 3~5 mm and the RMSE of registration between TLS and MLS point clouds is 2~4 cm. In addition, the proposed NSMS fitness function is more accurate and efficient than the existing Silva fitness function.

To accelerate the convergence of optimization, the ICP was integrated with GA for registration. The integrating strategy is that ICP is executed after GA evolves some generations and most of the individuals in the population are located in a narrowed search space. The combined method was also tested with the two test datasets. The optimizing efficiency of the integrated method was increased by about 50% compared with that of GA registration alone.

The proposed GA registration method can get globally optimal solutions in the search space without initial solutions and the feature extraction is also not required. However, in current algorithms of the first step, only the moving tree leave points can be removed out. The other moving points—e.g., the moving car point—can not be removed. A few moving points may not effect the registration. But it may not be true when there are too many moving objects in the scene. This special case would be considered and perfected in the follow-up work. Further research will mainly focus on extending the proposed method to automatically align multi-view TLS point clouds, multi-strip MLS point clouds or hybrid multi-view point clouds.

## Figures and Tables

**Table 1:** The important specifications of 3D Riegl VZ400 and 2D Riegl VUX [49].

	VZ400	VUX
Max range target reflectivity 80%	320 m	420 m
Max range target reflectivity 10%	120 m	150 m
Accuracy	5 mm@100 m	5 mm@100 m
Horizontal angular step width/Scan speed	0.0024°~0.5°	10–250 scan/s
Vertical angular step width	0.0024°~0.288°	0.0036°~0.3°
Laser Beam Divergence	0.3 mrad	0.5 mrad

**Table 2:** The comparison of the two test datasets.

	Data Set 1		Data Set 2	
	Source	Target	Source	Target
Horizontal angular step width/Scan speed	0.015°	0.015°	0.15°	200 scan/s
Horizontal resolution	2.6 cm@100 m	2.6 cm@100 m	5.2 cm@20 m	5–6 cm
Vertical angular step width	0.015°	0.015°	0.15°	0.3°
Vertical resolution	2.6 cm@100 m	2.6 cm@100 m	5.2 cm@20 m	5.2 cm@10 m
point count	one hundred million		ten million	
Accuracy	millimeter level		centimeter level	

**Table 3:** The specified algorithm parameters of the evaluation of the GA registration.

*D_max_*	100 m	*V_g_*	2.5 cm	*C*	0.05	*p_T_*	5%	*p_S_*	tested
*M*	100	*P_c_*	0.9	*P_m_*	0.1	*MAX_g_*	300	*MAX_b_*	20

**Table 4:** The results of selection of matching points without normal space sampling.

Datasets	Point Clouds	Remained Points (%)	Running Time (s)
Data set 1	*S*	11.56	155
	*T*	11.42	100
Data set 2	*S*	19.46	20
	*T*	58.38	31

**Table 5:** The accuracy and efficiency of different fitness functions.

Datasets	Fitness Functions	RMSEs (mm)			Number of Generations			Mean Optimizing Time (s)
		Min	Max	Mean	Min	Max	Mean	
Dataset 1	Silva	4.2	5.5	4.8	130	219	171	250
	NSMS	3.4	4.9	4.0	119	222	160	199
Dataset 2	Silva	61.1	97.9	75.1	107	224	162	202
	NSMS	22.9	36.1	28.8	93	242	154	163
